# Cost‐effective analysis of teclistamab and daratumumab versus ciltacabtagene autoleucel in relapsed multiple myeloma

**DOI:** 10.1111/bjh.70607

**Published:** 2026-06-07

**Authors:** Sohaib Irfan, M. Abdullah Jamil, Muhammad Bilal Abid

**Affiliations:** ^1^ The Aga Khan University Medical College Karachi Pakistan; ^2^ Stem Cell Transplantation and Cellular Immunotherapy Program, Department of Hematology/Oncology, UMC TLC2 Foundation Cancer Center Texas Tech University Health Science Center, School of Medicine Lubbock Texas USA

**Keywords:** bispecific antibody, CAR‐T‐cell therapy, cost‐effectiveness, multiple myeloma

To the Editor,

The recent results from the randomized, phase III MajesTEC‐3 trial showed a superior progression‐free survival (PFS) (hazard ratio, 0.17; 95% confidence interval [95% C.I.], 0.12–0.23; *p* < 0.001) of teclistamab (anti‐B‐cell maturation antigen [BCMA]‐targeted bispecific antibody) and daratumumab (anti‐CD38 monoclonal antibody) combination (tec/dara) in relapsed/refractory multiple myeloma (RRMM) compared with the standard of care (SOC), daratumumab plus dexamethasone combined with pomalidomide (DPd) or bortezomib (DVd) in second line and beyond (2L+) setting.^1^, ^2^ This is the same setting that currently also has a commercially available BCMA‐directed chimeric antigen receptor (CAR) T‐cell approval, ciltacabtagene autoleucel (cilta‐cel).^3^

Given the potential cost differences between a one‐time CAR‐T infusion, which carries unique access and logistical barriers, and continuous bispecific antibody therapy, which poses long‐term infection risks, we conducted a cost‐effectiveness analysis of tec/dara versus cilta‐cel from a United States (US) payers' perspective.^4^, ^5^

A partitioned survival model (PSM) was developed to evaluate the cost‐effectiveness of tec/dara (MajesTEC‐3) versus cilta‐cel (CARTITUDE‐4) for the treatment of patients with RRMM who received one to three prior lines of therapy (pLOT). Survival data using the respective clinical trials were extracted and compared. Baseline patient characteristics across both trials are summarized in Table S4. Both cohorts were broadly comparable in key prognostic features. Although notable differences included a higher rate of prior anti‐CD38 exposure in CARTITUDE‐4 (25.5% vs. 5.2%) and a universal lenalidomide‐refractory requirement in CARTITUDE‐4, reflecting a more refractory population. PFS for MajesTEC‐3 was modelled using the reported 36‐month PFS of 83.4%, extrapolated via exponential distribution. Costs were estimated in 2025 US dollars and analysis was performed from the perspective of a US payer. Our base‐case analysis capped teclistamab costs at 48 months to account for potential payer‐imposed constraints since the MajesTEC‐3 protocol permits treatment until progression. Future costs and health outcomes were discounted at 3% annually.^6^ The model simulates patient outcomes over a 10‐year time horizon, utilizing monthly cycles to capture rapid disease dynamics, as a 10‐year horizon captures the vast majority of the projected life expectancy for this late‐line RRMM population.

To execute this, the model tracks three mutually exclusive health states: PFS, progressed disease (PD), and death. Transition probabilities were derived from the reported clinical trial data. For the MajesTEC‐3 arm, the survival curve was fitted to the reported 36‐month PFS of 83.4% and the derived 12‐month PFS of 91.8%, consistent with the trial's hazard ratio of 0.17 relative to the standard of care.

For extrapolation, long‐term survival was modelled using parametric survival analysis. An exponential distribution was fitted to the reported 12‐month landmark survival points to project outcomes over the 10‐year horizon (Figure 1). The rationale for selecting the exponential distribution and its superiority over other multi‐parametric models is detailed in supplementary methods. To further validate this approach, Weibull (shape = 0.8) and log‐normal (sdlog = 1) distributions were explored as scenario analyses, yielding incremental cost‐effectiveness ratios (ICERs) of $296 358 and $358 631 per quality‐adjusted life year (QALY), respectively, both exceeding the base‐case estimate. This confirms that the exponential model represents the most conservative and favourable assumption for tec/dara (Table S5). Given that the median overall survival (OS) was not reached in the MajesTEC‐3 trial, we applied a conservative ‘shifted survival’ assumption. Details related to OS estimation and modelling based on PFS are provided in supplementary methods. Teclistamab costs were calculated based on the monthly recurring price ($28 158).^7^ Real‐world discontinuation patterns and payer restrictions were reflected in the base case analysis by capping the duration of teclistamab treatment at 48 months (4 years), despite the ‘until progression’ label in the trial protocol. Cilta‐cel was modelled as a one‐time cost of $465 000 plus an inpatient administration cost of $69 321.^8^

**FIGURE 1:**
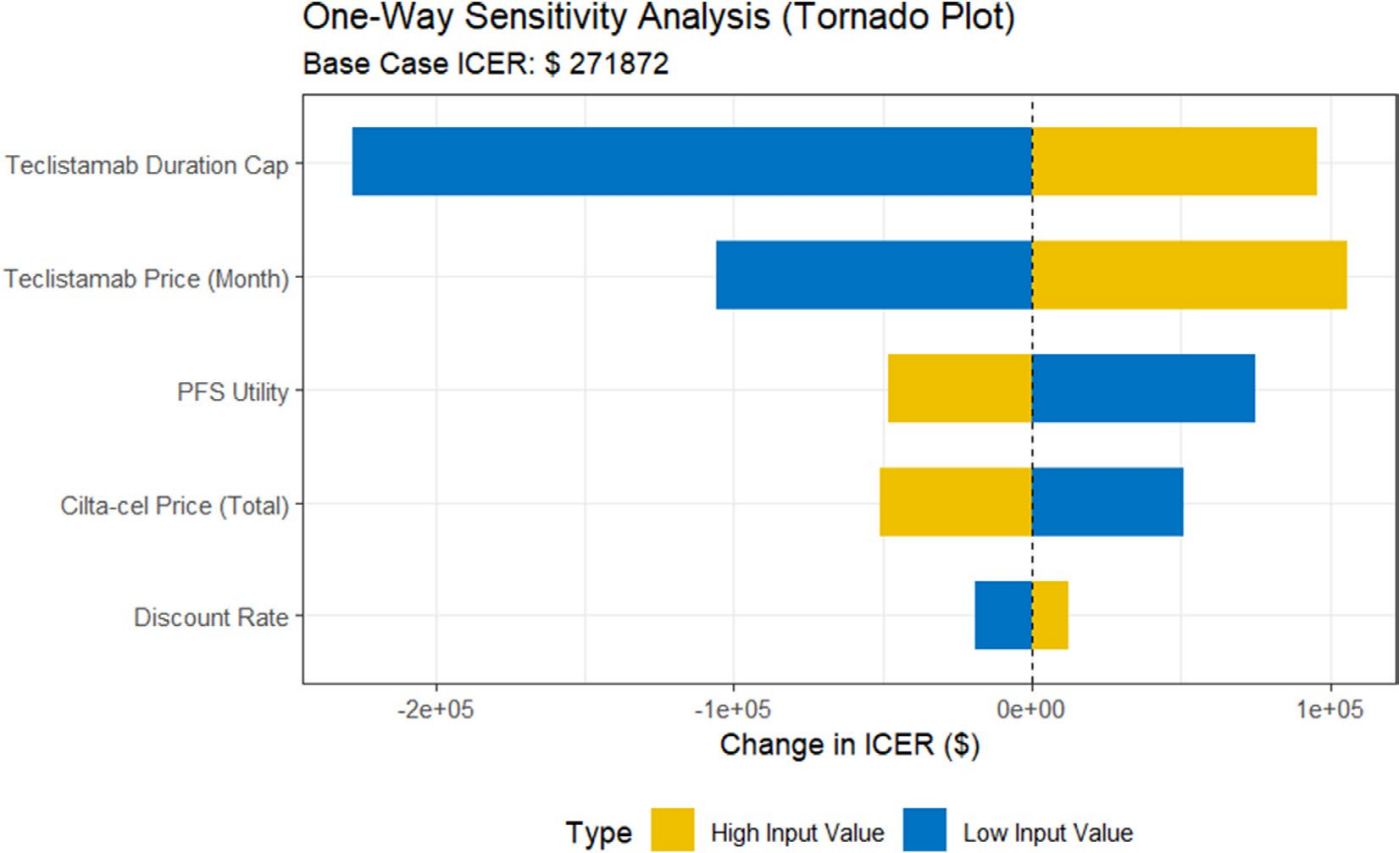
Modelled progression‐free survival (PFS) over a 10‐year horizon. Comparison of extrapolated progression‐free survival curves for teclistamab‐daratumumab (MajesTEC‐3) versus ciltacabtagene autoleucel (CARTITUDE‐4). Curves were generated using exponential parametric fits to simulate long‐term outcomes beyond the trial follow‐up periods.

Costs for managing adverse events (AE) were calculated as a weighted average based on incidence rates. For the MajesTEC‐3 arm, AE costs were adjusted to reflect the safety profile reported in the primary analysis. Drug acquisition and administration costs, applied to each treatment arm, are detailed in Table S1. Core structural assumptions, including discount rates for costs and outcomes, time horizon, cycle length and willingness‐to‐pay threshold, are presented in Table S2. Parameter values, uncertainty ranges and probability distributions used in deterministic and probabilistic sensitivity analyses are given in Table S3. PSM was chosen to directly utilize trial‐based survival partitions (PFS and OS) without requiring the complex transition state assumptions of a Markov model, which can introduce its own biases when patient‐level data isn't available.

To capture health‐related quality of life, utility values of 0.82 for the progression‐free state and 0.64 for the PD state were derived from published literature.^9^ A one‐time utility decrement of 0.10 was applied to the first cycle to account for the transient quality‐of‐life impact of severe adverse events (e.g. cytokine release syndrome [CRS], neurotoxicity).

To assess the robustness of the model assumptions, we performed both deterministic and probabilistic sensitivity analyses. A deterministic one‐way sensitivity analysis evaluated significant drivers of the ICER. Parameters variations included drug costs, discount rates, utility values and the treatment duration cap for teclistamab (Figure S1). A scenario analysis incorporating 80% real‐world adherence to teclistamab, compared with the 97.1% median relative dose intensity observed in the MajesTEC‐3 trial, yielded an ICER of $164 474 per QALY. This remained above the $150 000 threshold and confirms that the conclusion is robust even under conservative adherence assumptions. The base‐case results were derived from a probabilistic analysis using 1000 Monte Carlo simulations to account for simultaneous uncertainty across all model parameters. While 1000 iterations were utilized, model convergence was confirmed by the stability of the cost‐effectiveness scatterplot and the mean ICER, which showed negligible fluctuation with additional iterations during internal testing. Cost parameters were sampled from gamma distributions, while probabilities and utilities were sampled from Beta distributions to generate a cost‐effectiveness acceptability curve (CEAC) across a range of willingness‐to‐pay (WTP) thresholds (Figures S2 and S3).

The primary outcomes calculated were total costs, total QALYs and the ICER. The intervention was considered cost‐effective if the ICER fell below a willingness‐to‐pay threshold of $150 000 per QALY.^10^

All the analysis was conducted in R version 4.4.2. Full methodological details and internal validation procedures are described in supplementary methods.

Following these parameters, in the base‐case analysis over a 10‐year horizon, the MajesTEC‐3 tec/dara regimen incurred a total cost of $1 122 707 and generated 5.219 QALYs. In comparison, cilta‐cel was associated with a total cost of $557 021 and yielded 3.125 QALYs. Consequently, tec/dara was associated with an additional cost of $565 685.53 and a corresponding gain of 2.094 QALYs. At a WTP threshold of $150 000 per QALY, tec/dara was not cost‐effective relative to cilta‐cel.

Further exploration via deterministic one‐way sensitivity analysis demonstrated that teclistamab treatment duration cap is the most influential driver of ICER. Varying the duration cap produced the widest range of outcomes, from an ICER of $44 016.60 to $367 572.90 per QALY (Figure S1). Importantly, the lower bound of this range indicates that a restriction on treatment duration could make tec/dara regimen cost‐effective at the $150 000 threshold. Other significant parameters included ‘PFS utility values’, which yielded a range of $223 543.30–$346 860.00 per QALY, and the monthly price of teclistamab, influencing ICERs ranging from $166 235.10 to $377 508.10 per QALY. The model was relatively robust to changes in the discount rate (Table 1).

**TABLE 1:** One‐way sensitivity analysis of key model drivers.

Parameter	ICER at low input ($/QALY)	ICER at high input ($/QAL)	Range ($)
Teclistamab price (monthly)	166 235.10	377 508.10	211 273.04
Cilta‐cel price (total)	322 897.50	220 846.20	102 051.31
PFS utility	346 860.00	223 543.30	123 316.71
Discount rate	252 672.50	283 921.50	31 248.96
Teclistamab duration cap	44 016.60	367 572.90	323 556.26

Abbreviations: ICER, incremental cost‐effectiveness ratio; PFS, progression‐free survival; QALY, quality‐adjusted life year.

Moreover, the probabilistic base‐case analysis confirmed the stability of our findings, with 1000 simulations demonstrating a consistent cost‐effectiveness profile (Figure S2). At lower WTP thresholds, cilta‐cel exhibited a higher probability for the preferred therapy. MajesTEC‐3 demonstrated a low likelihood of cost‐effectiveness, with cilta‐cel remaining the preferred strategy in the majority of simulations at the standard WTP threshold of $150 000 per QALY. Tec/dara only became the more likely cost‐effective option at substantially higher WTP thresholds that exceed commonly accepted benchmarks (Figure S3). While modelled PFS curves consistently showed higher survival for MajesTEC‐3 over the 10‐year horizon, the substantial cumulative costs associated with prolonged therapy offset these clinical benefits within the current economic framework (Figure 1).

We acknowledge limitations in our approach. First, while our partitioned survival model directly utilizes trial‐based survival partitions, it does not explicitly incorporate the complex sequential treatment pathways that are increasingly being utilized in the RRMM landscape. The fixed 12‐month post‐progression survival lag is consistent with real‐world data from the LocoMMotion study. The study reported a median OS of 13.8 months and a median PFS of 4.6 months in triple‐class exposed patients with RRMM, implying a post‐progression survival of approximately 9.2 months in a more heavily pretreated population than ours.^11^ This supports 12 months as a conservative estimate for our 1–3 prior LOT setting. Sensitivity analyses across 6–18 months yielded ICERs of $256 240 to $285 596 per QALY, confirming minimal structural impact on conclusions (Table S5). As this analysis relies on a cross‐trial comparison between MajesTEC‐3 and CARTITUDE‐4, differences in patient populations and trial conduct may introduce selection bias. This is an inherent limitation when direct head‐to‐head, randomized evidence is unavailable. Furthermore, emerging evidence suggests that prior BCMA‐directed bispecific antibody therapy significantly limits the efficacy of subsequent CAR‐T‐cell therapy.^12^ This is an asymmetry in post‐progression treatment options that our model does not capture and that would further increase the ICER for tec/dara if formally incorporated. Finally, although our 10‐year time horizon captures the vast majority of life expectancy for this late‐line population, it deviates from a lifetime horizon and may inherently limit the capture of extreme long‐term uncertainties.

Taken together, our cost‐effectiveness analysis balances the survival benefits of tec/dara against the substantial economic challenges compared with CAR‐T therapy at current pricing. Notably, while modelled PFS outcomes were superior for tec/dara; the cumulative costs of continuous therapy drive the ICER well above the $150 000 per QALY threshold.^10^ The findings were robust in the sensitivity analysis, consistent with the potential economic value of fixed‐duration treatment approaches. Fixed‐duration bispecific antibody platforms and response‐adapted treatment discontinuation hold promise for balancing the regimen's cost with its survival benefits. As RRMM treatment landscape evolves, balancing the superior accessibility of bispecific antibodies against the cost efficiency of one‐time cellular therapies will be paramount for sustainable and equitable healthcare delivery.

## AUTHOR CONTRIBUTIONS

**Sohaib Irfan:** Data curation; formal analysis; methodology; writing – review and editing; writing – original draft. **M. Abdullah Jamil:** Data curation; writing – review and editing; writing – original draft. **Muhammad Bilal Abid:** Conceptualization; writing – review and editing; supervision; writing – original draft.

## CONFLICT OF INTEREST STATEMENT

The authors declare no competing financial interests.

## Supporting information


**Figure S1:** Tornado diagram of one‐way sensitivity analysis.
**Figure S2:** Cost‐effectiveness acceptability curve (CEAC).
**Figure S3:** Probabilistic sensitivity analysis (PSA) scatterplot.
**Table S1:** Base case cost inputs and resource utilization.
**Table S2:** Core structural model assumptions.
**Table S3:** Parameters for probabilistic and deterministic sensitivity analyses.
**Table S4:** Data extracted from published primary analyses of MajesTEC‐3 (Costa et al., NEJM 2026) and CARTITUDE‐4 (San‐Miguel et al., NEJM 2023).
**Table S5:** Scenario and sensitivity analyses for the cost‐effectiveness of teclistamab‐daratumumab versus ciltacabtagene autoleucel in relapsed/refractory multiple myeloma.

## Data Availability

All model inputs and parameters used in this analysis are included in the manuscript and supplementary online content. Original clinical data were extracted from publicly available clinical trial publications as cited. The code used for analysis is available from the corresponding author upon request.
