# Supplementary material for: Cost‐effective analysis of teclistamab and daratumumab versus ciltacabtagene autoleucel in relapsed multiple myeloma
Source: Br J Haematol. 2026 Jun 7;209(2):778–81. doi: 10.1111/bjh.70607 (PMC13462186; doi:10.1111/bjh.70607)
Supplement: Supplementary file 1 [file BJH-209-778-s001.docx]

**Supplementary**

**Cost-effective Analysis of Teclistamab and Daratumumab Versus** **Ciltacabtagene autoleucel in Relapsed Multiple Myeloma**

Sohaib Irfan, MBBS^1^; M. Abdullah Jamil, MBBS^1^; Muhammad Bilal Abid, MD, MS**^†^**^2^

^1^The Aga Khan University Medical College, Karachi, Pakistan.

^2^Texas Tech University Health Science Center School of Medicine, UMC Cancer Center, Lubbock, TX.

**Supplementary Methods**

**eFigure 1: Tornado Diagram of One-Way Sensitivity Analysis**

**eFigure 2: Cost-Effectiveness Acceptability Curve (CEAC)**

**eFigure 3: Probabilistic Sensitivity Analysis (PSA) Scatterplot**

**eTable 1: Base Case Cost Inputs and Resource Utilization**

**eTable 2: Core Structural Model Assumptions**

**eTable 3: Parameters for Probabilistic and Deterministic Sensitivity Analyses**

**eTable 4: Data extracted from published primary analyses of MajesTEC-3 (Costa et al., NEJM 2026) and CARTITUDE-4 (San-Miguel et al., NEJM 2023).**

**eTable 5: Scenario and Sensitivity Analyses for the Cost-Effectiveness of Teclistamab-Daratumumab versus Ciltacabtagene Autoleucel in Relapsed/Refractory Multiple Myeloma**

**Supplementary Methods**

**Survival Extrapolation**

Exponential distribution was selected as it allows for robust extrapolation from the 12-month landmark survival data reported in MajesTEC-3. This approach is safer than using multi-parameter models like Weibull or Log-normal when extracting from summary trial data, as it assumes a constant hazard rate and provides a clinically conservative, plausible long-term tail for relapsed, refractory multiple myeloma (RRMM).

**Overall Survival Estimation and Relationship to Progression-Free Survival**

Overall survival (OS) is derived by adding a fixed post-progression survival (PPS) constant (12 months) to the PFS curve, ensuring internal consistency between the two health states. OS was modeled as a function of PFS with a fixed PPS lag of 12 months, ensuring that mortality accounts for disease progression.

**Statistical Analysis and Internal Model Validation**

All the analysis was conducted in R version 4.4.2 using dplyr, tidy, and ggplot packages. Internal validity was established through "white-box" testing, confirming that the sum of the health state traces (Progression-Free, Progressed, and Dead) equaled 1.0 at every cycle and that cost accumulation logic accurately reflected the defined treatment stopping rules. The analysis followed standard reporting guidelines for economic evaluations, prioritizing the ICER as the primary metric for decision-making. While the model focuses on the index therapies, subsequent lines of therapy were assumed to be balanced between arms or captured within the post-progression cost/utility estimates.

**eFigure 1: Tornado Diagram of One-Way Sensitivity Analysis**

**Abbreviations:** **ICER**: incremental cost-effectiveness ratio

**eFigure 2: Cost-Effectiveness Acceptability Curve (CEAC)**

**Abbreviations:** **WTP:** willingness to pay

**eFigure 3: Probabilistic Sensitivity Analysis (PSA) Scatterplot**

**Abbreviations:** **QALY:** Quality-Adjusted Life Years

**eTable 1: Base Case Cost Inputs and Resource Utilization**

| **Cost Category** | **Cost Item** | **Unit Cost (USD)** | **Frequency** | **Applied To Arm** |
| --- | --- | --- | --- | --- |
| **Drug** | Teclistamab | 28,158 | Monthly | MajesTEC-3 |
| **Drug** | Cilta-cel | 465,000 | One-time | CARTITUDE-4 |
| **Administration** | CAR-T Inpatient Stay | 69,321 | One-time | CARTITUDE-4 |
| **AE Management** | CRS (Grade 1-2) | 27,615 | Per Event | Both |
| **AE Management** | CRS (Grade ≥3) | 99,894 | Per Event | Both |
| **AE Management** | Neurotoxicity (Grade 1-2) | 21,164 | Per Event | CARTITUDE-4 |
| **AE Management** | Neurotoxicity (Grade ≥3) | 51,158 | Per Event | CARTITUDE-4 |
| **Abbreviations:** **AE:** Adverse event management, **CAR-T:** Chimeric Antigen Receptor T cell, **CRS:** Cytokine Release Syndrome, **USD:** United States Dollar | | | | |

**eTable 2: Core Structural Model Assumptions**

| **Parameter** | **Value** | **Distribution** | **Lower Bound** | **Upper Bound** |
| --- | --- | --- | --- | --- |
| **Discount Rate Costs** | 0.03 | Fixed | 0 | 0.05 |
| **Discount Rate Outcomes** | 0.03 | Fixed | 0 | 0.05 |
| **Time Horizon (Months)** | 240 | Fixed | 120 | Lifetime |
| **Cycle Length** | 1 | Fixed | NA | NA |
| **WTP Threshold** | $150,000 | Fixed | $50,000 | 200,000 |
| **Abbreviations: WTP:** willingness to pay, **NA:** Not applicable | | | | |

**eTable 3: Parameters for Probabilistic and Deterministic Sensitivity Analyses**

| **Parameter** | **Base Case** | **Low** | **High** | **Distribution** |
| --- | --- | --- | --- | --- |
| **Discount Rate Costs** | 0.03 | 0 | 0.05 | Fixed |
| **Discount Rate Outcomes** | 0.03 | 0 | 0.05 | Fixed |
| **Cost Teclistamab Month** | $28,158 | $22,526 | $33,790 | Gamma |
| **Cost CiltaCel OneTime** | $465,000 | $372,000 | $558,000 | Gamma |
| **Cost CAR-T Inpatient** | $69,321 | $55,457 | 83,185 | Gamma |
| **Utility PFS** | 0.82 | 0.66 | 0.98 | Beta |
| **Utility PD** | 0.64 | 0.51 | 0.77 | Beta |
| **Disutility CRS Grade3** | -0.1 | -0.15 | -0.05 | Beta |
| **Patient Weight kg** | 75 | 60 | 90 | Normal |
| **Time Horizon Years** | 20 | 10 | 30 | Fixed |
| **Abbreviations: Ciltacel:** ciltacabtagene, **CAR-T:** Chimeric Antigen Receptor T-Cell, **PFS:** Progression Free Survival, **PD:** Disease progression, **CRS:** Cytokine Release Syndrome | | | | |

**eTable 4: Data extracted from published primary analyses of MajesTEC-3 and CARTITUDE-4**

| **Characteristic** | **MajesTEC-3 Tec/Dara (N=291)** | **CARTITUDE-4 Cilta-cel (N=208)** |
| --- | --- | --- |
| Median age, years (range) | 64 (36–88) | 61.5 (27–78) |
| Male sex, % | 53.6 | 55.8 |
| ECOG PS 0, % | 57.4 | 54.8 |
| ECOG PS 1, % | 37.1 | 44.7 |
| ECOG PS 2, % | 5.5 | 0.5 |
| ISS Stage I, % | 62.5 | 65.4 |
| ISS Stage II, % | 29.2 | 28.8 |
| ISS Stage III, % | 8.2 | 5.8 |
| Median prior lines of therapy (range) | 2 (1–3) | 2 (1–3) |
| Prior anti-CD38 antibody exposure, % | 5.2 | 25.5 |
| Lenalidomide-refractory, % | 84.9 | 100 (entry criterion) |
| High-risk cytogenetics, % | 36.5 | 35.3 |
| Median time from diagnosis, years | 3.7 | 3 |
| **Abbreviations**: **ECOG PS:** Eastern Cooperative Oncology Group Performance Status, **ISS:** International Staging System | | |

**eTable 5: Scenario and Sensitivity Analyses for the Cost-Effectiveness of Teclistamab-Daratumumab versus Ciltacabtagene Autoleucel in Relapsed/Refractory Multiple Myeloma**

| **Scenario** | **Parameter Value** | **ICER ($/QALY)** | **Cost-Effective at $150,000 Threshold** |
| --- | --- | --- | --- |
| Base Case | Exponential, 48m cap, 12m PPS lag | $270,103 | No |
| Alternative Survival Distributions |  |  |  |
| Weibull distribution | Shape = 0.8 | $296,358 | No |
| Log-normal distribution | SDlog = 1.0 | $358,631 | No |
| Post-Progression Survival Lag |  |  |  |
| PPS lag, short | 6 months | $256,240 | No |
| PPS lag, base case | 12 months | $270,103 | No |
| PPS lag, extended | 18 months | $285,596 | No |
| Real-World Adherence |  |  |  |
| Adherence scenario | 80% of monthly dose | $164,474 | No |
| Teclistamab Duration Cap |  |  |  |
| Short cap | 24 months | $42,248 | Yes |
| Intermediate cap | 36 months | $162,726 | No |
| Base case cap | 48 months | $270,103 | No |
| Extended cap | 60 months | $365,805 | No |
| No cap | Until progression | $709,080 | No |
| **Abbreviations: ICER:** Incremental Cost-Effectiveness Ratio**, QALY:** Quality-Adjusted Life Year**, PPS:** Post-Progression Survival**, SDlog:** Standard Deviation (log scale) | | | |
